# Unbelief

**Published:** 1890-08-16

**Authors:** 


### Words of Consolation.

### UNBELIEF.

We read in St. Matthew's Gospel that when our Lord went into His own country and taught at Capernaum, that the people were offended at Him and asked in derision, Where did this man get his wisdom from, and how does he do his mighty works? Is not this the carpenter's son? Don't we know his mother, and are not his relations living with us? So "He did not many mighty works there because of their unbelief."

We must not suppose that our Lord refused to use His power in order to punish His fellow-countrymen for their unkind taunts; that would be to make Him a vindictive God, whereas, on the contrary, He is full of mercy and loving kindness, even to those who hate Him. No, there was a far deeper reason, and St. Mark lets us into the secret when he says, "He could there do no mighty works, save that He healed a few sick folk, because of their unbelief."

How strange this is, you say. Have I not been always taught that Christ is God, and yet you tell me it was impossible for Him to do any mighty works at Capernaum? True on both points. Christ is God, but He cannot act contrary to His nature and the laws of His being. He is almighty omnipotent; He is also a God that cannot lie; therefore He will not swerve from His commandments.

God made man a free agent, to glorify Him by a free service, and the service which He requires is that we shall accept the workings of His grace. We are free to accept or reject, but if we accept, it is by what is called faith. By faith we do not mean a mere belief in the fact that there is a God; the devils do that and tremble, but we must have a saving faith in our hearts, as God will force no one to be saved. He will draw us by the cords of His love. Our Lord says, "No man can come to Me except the Father which sent Me draw him." So, then, when we feel this drawing we must yield ourselves with trustfulness to it, and though our faith may at first be weak, yet we have the same promise that Christ gave to the lunatic child's father, "All things are possible to him that believeth," and we should respond with the cry of the despairing parent, "Lord, I believe, help Thou mine unbelief."

God is hindered now, as in old times, by our unbelief. He cannot do the mighty works for our benefit which He longs to do. We can but own that there is a God, since all our surroundings speak loudly and forcibly of an almighty power. We may even go farther and say, "Christ died for me," but there we stop; a few sick folk only are healed, brought to Him by the prayers and tears of loving hearts, who feel for the sufferings of our fellow-men. It is the mighty work of salvation of souls that is prevented by our faithless hearts. It is our souls, sick unto death, that want healing. God, therefore, stoops from His throne of glory and comes to us in His sacraments of grace. Instead of thankfully accepting these blessings and making them our own, we say, "How can a man be born again? Will a little water suffice to cleanse our souls?" Or, with the men of Capernaum, "Is not this just bread and wine! whence, then, hath it all this power?" and we, too, are offended. It is this utter faithlessness which prevents our making progress in our struggles against sin; we want to understand everything; we cannot trust God to do His mighty works in His own way; we want to do all ourselves and in the way which we think best. Have we thought of the words, "No man can deliver his own soul?" God's grace is always ready; it presses upon us, only we must yield ourselves to it. We are like shipwrecked travellers who are afraid to trust themselves to the water lest they should sink. The water is ready to support them if they will only cast themselves on it boldly. But though God's grace is around us everywhere, yet our want of faith mars His work. Oh! all but shipwrecked soul! Awake in time to the sense of your own helplessness, and take His gracious promises to yourself. Let it not be said of you, He could do no mighty work in you because of your unbelief.

